# Tumor-Infiltrating T Cells Correlate with NY-ESO-1-Specific Autoantibodies in Ovarian Cancer

**DOI:** 10.1371/journal.pone.0003409

**Published:** 2008-10-15

**Authors:** Katy Milne, Rebecca O. Barnes, Adam Girardin, Melanie A. Mawer, Nancy J. Nesslinger, Alvin Ng, Julie S. Nielsen, Robert Sahota, Eric Tran, John R. Webb, May Q. Wong, Darin A. Wick, Andrew Wray, Elissa McMurtrie, Martin Köbel, Steven E. Kalloger, C. Blake Gilks, Peter H. Watson, Brad H. Nelson

**Affiliations:** 1 Trev and Joyce Deeley Research Centre, BC Cancer Agency, Victoria, British Columbia, Canada; 2 Department of Biochemistry and Microbiology, University of Victoria, Victoria, British Columbia, Canada; 3 BC Cancer Agency, Victoria, British Columbia, Canada; 4 Department of Anatomical Pathology, Vancouver General Hospital, Vancouver, British Columbia, Canada; 5 Department of Pathology, University of British Columbia, Vancouver, British Columbia, Canada; 6 Department of Medical Genetics, University of British Columbia, Vancouver, British Columbia, Canada; University of California Los Angeles, United States of America

## Abstract

**Background:**

Tumor-infiltrating CD8+ T cells are correlated with prolonged progression-free and overall survival in epithelial ovarian cancer (EOC). A significant fraction of EOC patients mount autoantibody responses to various tumor antigens, however the relationship between autoantibodies and tumor-infiltrating T cells has not been investigated in EOC or any other human cancer. We hypothesized that autoantibody and T cell responses may be correlated in EOC and directed toward the same antigens.

**Methodology and Principal Findings:**

We obtained matched serum and tumor tissue from 35 patients with high-grade serous ovarian cancer. Serum samples were assessed by ELISA for autoantibodies to the common tumor antigen NY-ESO-1. Tumor tissue was examined by immunohistochemistry for expression of NY-ESO-1, various T cell markers (CD3, CD4, CD8, CD25, FoxP3, TIA-1 and Granzyme B) and other immunological markers (CD20, MHC class I and MHC class II). Lymphocytic infiltrates varied widely among tumors and included cells positive for CD3, CD8, TIA-1, CD25, FoxP3 and CD4. Twenty-six percent (9/35) of patients demonstrated serum IgG autoantibodies to NY-ESO-1, which were positively correlated with expression of NY-ESO-1 antigen by tumor cells (r = 0.57, p = 0.0004). Autoantibodies to NY-ESO-1 were associated with increased tumor-infiltrating CD8+, CD4+ and FoxP3+ cells. In an individual HLA-A2+ patient with autoantibodies to NY-ESO-1, CD8+ T cells isolated from solid tumor and ascites were reactive to NY-ESO-1 by IFN-γ ELISPOT and MHC class I pentamer staining.

**Conclusion and Significance:**

We demonstrate that tumor-specific autoantibodies and tumor-infiltrating T cells are correlated in human cancer and can be directed against the same target antigen. This implies that autoantibodies may collaborate with tumor-infiltrating T cells to influence clinical outcomes in EOC. Furthermore, serological screening methods may prove useful for identifying clinically relevant T cell antigens for immunotherapy.

## Introduction

Epithelial ovarian cancer (EOC) is a challenging disease that affects more than 190,000 women worldwide each year (International Agency for Research on Cancer). The high mortality rate is attributed to the fact that most patients are diagnosed with disseminated disease, often with extensive ascites. Standard treatment involves cytoreductive surgery followed by taxane- and platinum-based chemotherapy [1]. Over 80% of patients are highly responsive to frontline treatment, but 60–70% experience disease recurrence within 2–5 years and ultimately succumb to their disease [2], [3].

Despite these unfortunate statistics, 20–30% of EOC patients survive five years or more after diagnosis. Favorable prognostic factors include early stage, non-serous histology, low grade, good performance status, and optimal surgical debulking [4], [5]. In addition, several recent studies have shown a correlation between tumor-infiltrating CD3+CD8+ T cells and favorable outcomes [6], [7]. Zhang *et. al.* first reported that patients with CD3+ T cell infiltrates in tumor epithelium had increased progression-free and overall survival [8]. This has been confirmed by two other studies [9], [10], and two groups have extended this finding to the CD8+ T cell subset in particular [11], [12]. In addition, the presence of CD3+CD56+ T cells in ascites has been linked to platinum sensitivity [13]. These findings are in agreement with earlier studies showing a positive correlation between survival and expression of interferon-γ (IFN-γ) [14], [15], the IFN-γ receptor [16], IL-18 [17], and MHC class I [18], [19], all of which are characteristic of CD8+ T cell responses. In contrast, the presence of tumor-infiltrating CD25+FoxP3+ T cells in EOC is correlated with inferior survival [11], [20]–[22]. Thus, it appears that the balance of CD8+ effector T cells to CD25+FoxP3+ regulatory T cells is an important determinant of clinical outcomes in EOC.

In addition to tumor-infiltrating T cells, many EOC patients mount serum autoantibody responses to a variety of tumor antigens, including NY-ESO-1, HOXA7, Ep-CAM, HSP-90, MUC-1 and p53 [23]–[25]. In Type I diabetes and other autoimmune conditions, the development of autoantibody responses portends tissue infiltration and destruction by autoreactive T cells [26]. We therefore hypothesized that EOC patients may show a similar relationship between tumor-specific autoantibody responses and tumor-infiltrating lymphocytes. This hypothesis was tested in a cohort of 35 advanced stage, high grade serous EOC cases for which matched serum and tumor specimens were available. Using NY-ESO-1 as a test antigen, we demonstrate for the first time a correlation between tumor-specific autoantibodies and tumor-infiltrating T cells. Our findings raise the possibility that autoantibodies may play a role in the previously recognized relationship between tumor-infiltrating T cells and clinical outcomes in EOC.

## Results

### Study cohort

We investigated the relationship between tumor-specific autoantibodies and tumor-infiltrating lymphocytes using matched tumor and serum specimens from a retrospective cohort of 35 patients with high-grade serous EOC (Table 1). We elected to focus on a single histological subtype, as other subclasses of EOC exhibit distinct biological and clinical properties that might have confounded the analysis [27]. All blood samples were collected prior to surgery or chemotherapy, and all tumor specimens were obtained at the time of primary cytoreductive surgery prior to chemotherapy. Control blood samples were obtained from 60 age-matched women with no known personal history of cancer.

**Table 1:** Clinical characteristics of the retrospective patient cohort.

Age at surgery (years)	
Mean	61.93
Std dev	15.61
Range	22.52–90.99
Median	63.61
* Overall Survival (years)	
Mean	1.63
Std dev	0.703
Range	0–3.06
Median	1.69
Silverberg Grade	
1	0
2	10
3	23
Unknown	2
Stage	
I	4
II	3
III	19
IV	4
Unknown	5
Total number of evaluable patients	35

* There were no deaths due to causes other than ovarian cancer, therefore disease-specific and overall survival were equivalent.

### Analysis of tumor-infiltrating lymphocytes

**Figure 1:**
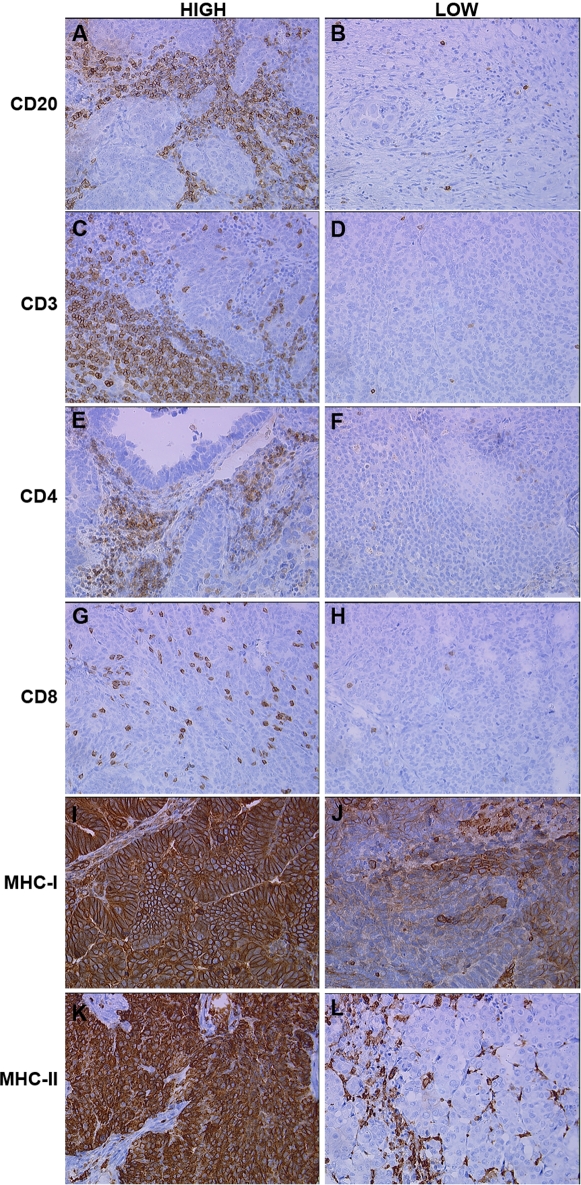
Immunohistochemical analysis of serous ovarian tumors showing cases with high (left) and low (right) scores for the following markers: (A,B) CD20; (C,D) CD3; (E,F) CD4; (G,H) CD8; (I,J) MHC Class I; and (K,L) MHC Class II.

Tumor-infiltrating lymphocytes were assessed by immunohistochemistry (IHC) using antibodies to a variety of immunological markers (Figure 1, Table 2). All raw IHC data can be found in Supplementary Tables S1 and S2. Only 23% (8/35) of evaluable tumors showed significant CD20+ B cell infiltrates. In contrast, 94% (33/35) of tumors had detectable CD3+ T cell infiltrates. Staining with antibodies to CD4 and CD8 revealed that 59% (20/34) and 69% (24/35) of evaluable tumors had significant CD4+ and CD8+ cellular infiltrates, respectively. CD4+ and CD8+ cells were strongly correlated (r = 0.69, p<0.0001). All evaluable tumors (27/27) expressed MHC class I to some degree, indicating they could theoretically present antigen to the infiltrating CD8+ T cells. Seventy-two percent (18/25) of tumors expressed MHC class II and hence could theoretically present antigen to CD4+ T cells.

**Table 2:** Primary antibodies used for immunohistochemistry.

Antigen	Clone	Supplier	Catalogue	Source	Concentration
NY-ESO-1	E978	Zymed	35-6200	Mouse	1/200
CD20	Polyclonal	Lab Vision	RB-9013	Rabbit	1/400
CD3	SP7	Lab Vision	RM-9107	Rabbit	1/150
CD4	4B12	Lab Vision	MS-1528	Mouse	1/10
CD8	SP16	Lab Vision	RM-9116	Rabbit	1/100
FoxP3	eBio7979	eBioscience	14-7979	Mouse	1/50
CD25	4C9	Lab Vision	MS-1088	Mouse	1/40
Granzyme B	Polyclonal	Abcam	ab4059	Rabbit	1/50
TIA-1	TIA-1	Abcam	ab2712	Mouse	1/50
MHC class I (A, B, C)	EMR8-5	MBL	D226-3	Mouse	1/500
MHC class II (DR, DP & DQ)	CR3/43	Affinty BioReagents	MA1-25914	Mouse	1/50
Pan-cytokeratin	PCK-26	Sigma	C1801	Mouse	1/300

**Figure 2:**
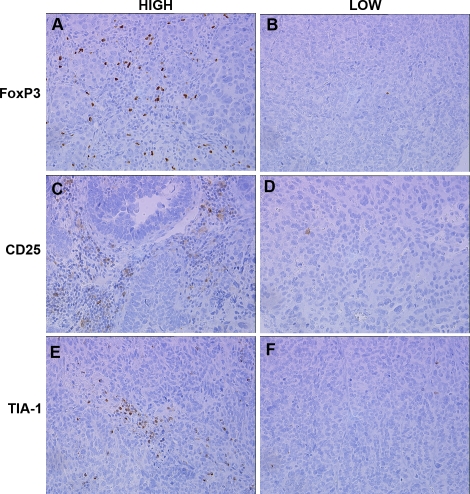
Immunohistochemical analysis of serous ovarian tumors showing cases with high (left) and low (right) scores for the following markers: (A,B) TIA-1; (C,D) FoxP3; and (E,F) CD25.

Since many tumors had dense CD8+ T cell infiltrates, we analyzed tissues for TIA-1 and Granzyme B, both of which are expressed by CD8+ cytotoxic T cells as well as natural killer cells [28]–[30]. Seventy-three percent (25/34) of tumors had significant TIA-1+ cellular infiltrates, which showed a positive correlation with CD8+ infiltrates (r = 0.83, p<0.0001) (Figure 2). In contrast, only 20% (7/35) of the tumors had significant Granzyme B+ cellular infiltrates (data not shown); as expected, 6 of the 7 positive tumors were also positive for CD8+ cells.

Tumors were also analyzed for cellular infiltrates expressing FoxP3, a marker of activated T cells and regulatory T cells [31]. Sixty-six percent (23/35) of evaluable tumors had significant FoxP3+ infiltrates (Figure 2), which were strongly correlated to CD4+ cells (r = 0.73, p<0.0001). We also evaluated expression of CD25, an additional marker of activated and regulatory T cells [32]. Fifty percent (17/34) of tumors had significant CD25+ cellular infiltrates (Figure 2), which were strongly correlated with CD4+ cells (r = 0.58, p = 0.0003) and FoxP3+ cells (r = 0.75, p<0.0001).

We further stratified T cell infiltrates according to their epithelial or stromal location within the tumor, as intraepithelial CD3+/CD8+ T cells in particular have been correlated with increased survival in EOC [8]–[12]. For this analysis, we first measured the tumor composition as defined by the epithelial∶stromal ratio in each tissue core and then calculated the density of T cells per unit of epithelium or stroma. As summarized in Table 3, the density of CD3+ T cells per unit of tumor epithelium ranged from 0–17, with a median of 4.6. In comparison, the density of CD3+ T cells per unit of tumor stroma ranged from 0–138, with a median of 16.3. In general, the density of CD3+ T cells in tumor epithelium and stroma were only weakly correlated (r = 0.34, p = 0.048), and there were many examples of tumors with dense CD3+ infiltrates in epithelium but not stroma, and conversely, in stroma but not epithelium. Similar to CD3+ cells, CD8+, CD4+, FoxP3+ and TIA-1+ cells were generally denser in tumor stroma than tumor epithelium (Table 3).

**Table 3:** Density of lymphocyte subsets in tumor stroma versus epithelium.

Marker	* Epithelial Density		* Stromal Density	
	Median	Range	Median	Range
CD3	4.6	0–17	16.3	0–138
CD8	1.8	0–17	6.6	0–49
CD4	1	0–13	4	0–123
FoxP3	1	0–9	7	0–74
CD25	1.1	0–25	1.1	0–29
TIA-1	1.4	0–13	4.5	0–33
Granzyme B	0	0–6	0	0–47
CD20	0	0–3	0	0–106

* All values are reported as cells per unit area defined by the Chalkley grid.

### Composition of tumor-infiltrating lymphocytes after neoadjuvant chemotherapy

The TMA used in the above analyses also contained an additional cohort of 15 tumors from women who, as part of a clinical trial, had undergone neoadjuvant platinum/taxane-based chemotherapy prior to their primary surgery. As with the 35-case cohort, these women had high-grade serous EOC. Tumors had been resected after three cycles of carboplatinum/taxol-based chemotherapy. Although the sample size was small, this provided a unique opportunity to evaluate the effects of chemotherapy on tumor-infiltrating lymphocytes. By most parameters, lymphocytic infiltrates were similar between treated and untreated tumors. However, treated tumors showed a uniform trend towards increased infiltration by all subsets of T cells assessed, and this increase was significantly higher for CD3+ (median 80 vs 35, p = 0.02) and CD8+ (median 78 vs 30, p = 0.013) cells (data not shown).

### Serum autoantibody responses to NY-ESO-1

**Figure 3:**
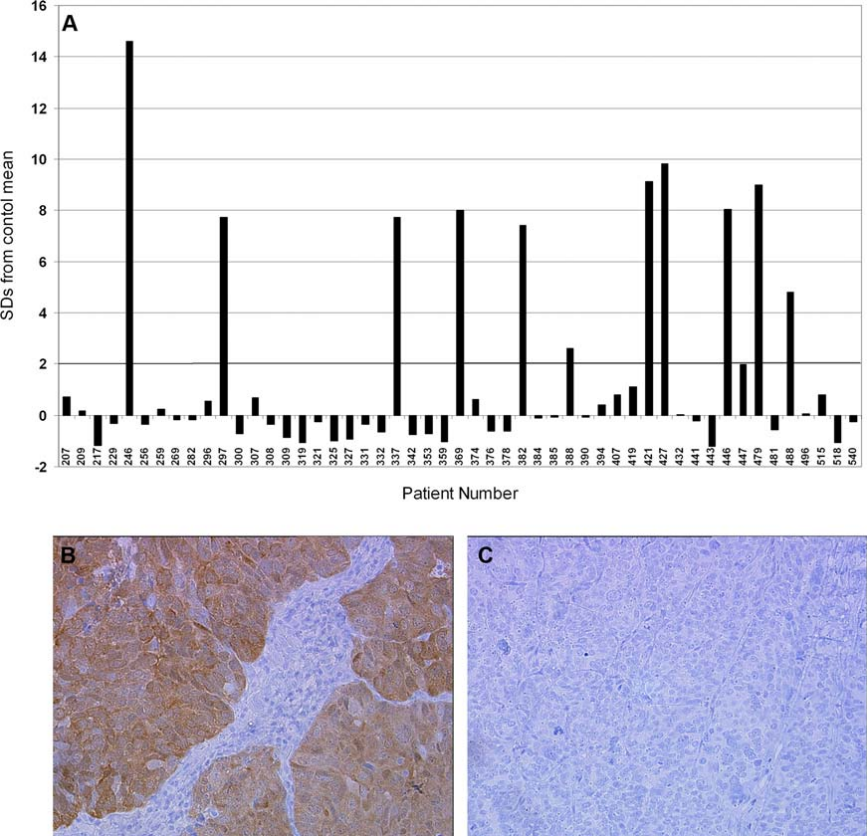
NY-ESO-1 serum autoantibodies and antigen expression in high-grade serous ovarian cancer. (A) Serum autoantibody responses to NY-ESO-1 in the 35-patient cohort. Autoantibody responses are reported as the number of standard deviations from the mean of 60 age- and gender-matched controls with no known personal history of cancer. (B,C) Immunohistochemical analysis of NY-ESO-1 expression in two representative serous ovarian tumors with high (B) and negative (C) expression of the antigen.

Although ovarian cancer patients demonstrate autoantibody responses to a broad repertoire of antigens [23]–[25], we focused on one of the most immunogenic antigens, NY-ESO-1 [24], [33], [34]. Sixty control sera were assayed for IgG autoantibodies to recombinant NY-ESO-1, and the mean and standard deviation of the OD values were calculated (Supplementary Tables S1 and S2). Individual sera were scored as positive if their OD value was equal to or greater than two standard deviations from the mean of control subjects. Consistent with published results, 26% (9/35) of ovarian cancer cases demonstrated IgG autoantibodies to NY-ESO-1, compared to only 5% (3/60) of controls (Figure 3A).

To determine whether autoantibody responses to NY-ESO-1 correlated with expression of the corresponding antigen, matched tumor specimens were analyzed by IHC for expression of NY-ESO-1. Of 34 evaluable tumors, 5 (14.7%) scored positive for NY-ESO-1 antigen (Figure 3B). All five of these cases were also positive for autoantibodies to NY-ESO-1. In contrast, 4 cases were positive for NY-ESO-1-specific autoantibodies but negative for NY-ESO-1 antigen expression.

### Correlations between autoantibody responses and lymphocytic infiltrates

We next investigated whether serum autoantibodies to NY-ESO-1 were correlated with tumor-infiltrating lymphocytes. For this analysis, we considered epithelial and stromal T cell infiltrates separately, as described above. We classified cases as being positive or negative for autoantibodies to NY-ESO-1 using a cut-point of two standard deviations from the mean of the control group (Supplementary Tables S1 and S2). By Mann Whitney t test, patients with autoantibodies to NY-ESO-1 had a significantly greater stromal density of CD8+ cells (p = 0.011), FoxP3+ cells (p = 0.013), and CD4+ cells (p = 0.026). Thus, autoantibodies to NY-ESO-1 showed a significant correlation to T cell infiltrates, especially in tumor stroma.

### Recognition of NY-ESO-1 by autoantibodies and tumor-infiltrating CD8+ T cells from the same patient

**Figure 4:**
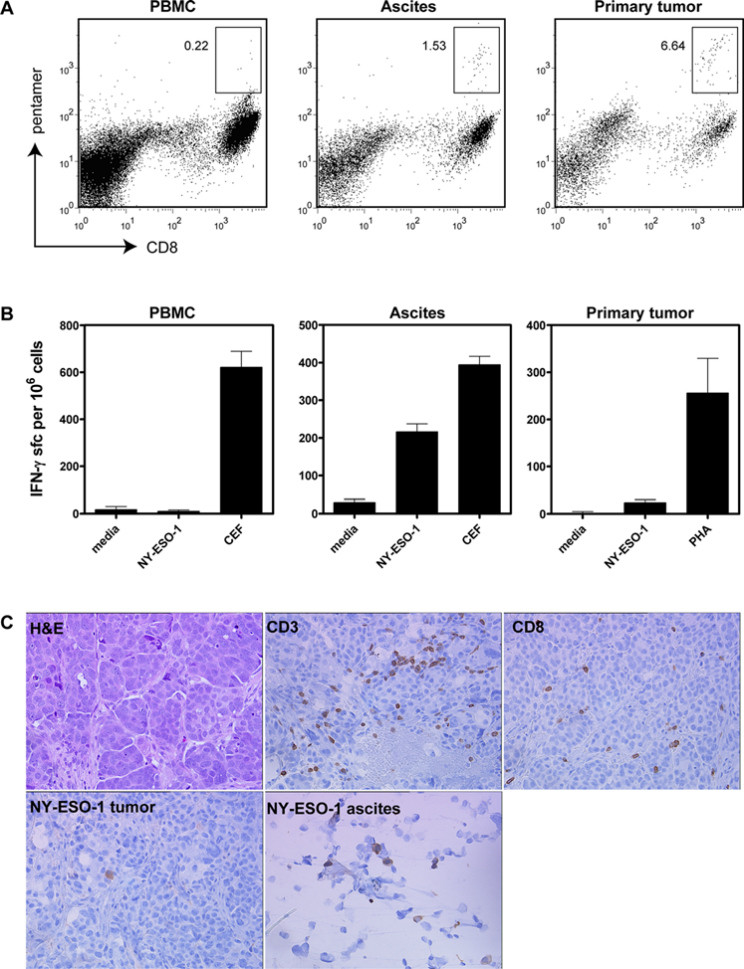
Analysis of the T cell response in a patient with autoantibodies to NY-ESO-1 showing the presence of NY-ESO-1 reactive CD8+ T cells in ascites and tumor despite the lack of NY-ESO-1 expression in solid tumor. (A) MHC class I pentamer analysis demonstrating enrichment of NY-ESO-1-specific CD8+ T cells in ascites and solid tumor compared to peripheral blood. The boxed areas and associated numbers represent the percentage of pentamer-positive cells relative to total CD8+ cells. (B) ELISPOT analysis of IFN-γ production by T cells after stimulation with an HLA-A2-binding peptide from NY-ESO-1. Data is presented as the number of IFN-γ-producing cells per 1×10^6^ bulk cells from the indicated tissue compartments. (C) Immunohistochemical analysis of tumor-infiltrating CD3+ and CD8+ T cells in tumor stroma, and expression of NY-ESO-1 antigen. While the solid tumor was negative for expression of NY-ESO-1, a fraction of cells from ascites were positive. The cellular fraction of ascites also contained cytokeratin-positive epithelial cells, presumably of tumor origin (data not shown).

The correlation between autoantibodies to NY-ESO-1 and tumor-infiltrating T cells suggested the possibility that tumor-infiltrating T cells may recognize NY-ESO-1 in seropositive patients. To address this issue, we collected blood, tumor and ascites specimens from a prospective cohort of 15 newly diagnosed serous EOC patients. Two patients were positive for serum autoantibodies to NY-ESO-1. Of these, one case (IROC013) was HLA-A2+, allowing T cells to be enumerated by flow cytometry with HLA-A2 pentamers loaded with a known CD8+ epitope from NY-ESO-1 (Figure 4A). NY-ESO-1-specific CD8+ T cells were rare in peripheral blood from this patient (0.22% of CD8+ cells), but were enriched in ascites and solid tumor (1.53% and 6.64% of CD8+ cells, respectively). Furthermore, T cells from ascites and solid tumor produced IFN-γ in response to NY-ESO-1 peptide (Figure 4B) but not control peptides derived from p53, HER-2/*neu* or WT-1 (data not shown). Indeed, in ascites the response to NY-ESO-1 was almost as strong as that seen to the CEF viral control peptides. Intriguingly, tumor tissue from this patient showed dense CD3+ and CD8+ T cell infiltration of tumor stroma but not epithelium (Figure 4C), similar to the pattern commonly seen with autoantibody-positive patients in the retrospective cohort. Despite mounting a strong humoral and T-cell response to NY-ESO-1, solid tumor from this patient stained negative for expression of NY-ESO-1 antigen; however, ascites from this patient contained NY-ESO-1-positive cells (Figure 4C).

## Discussion

Using matched serum and tumor specimens from 35 patients with high-grade serous EOC, we have demonstrated a correlation between tumor-infiltrating T cells and tumor-specific autoantibodies. To our knowledge, this is the first study to identify such a correlation in any human cancer. Specifically, the presence of NY-ESO-1-specific IgG autoantibodies in serum correlated with infiltration of tumor stroma by cells expressing CD8, CD4 and FoxP3. Moreover, in an individual patient with autoantibodies to NY-ESO-1, corresponding ascites and solid tumor specimens were shown to be enriched for NY-ESO-1-reactive CD8+ T cells, as assessed by MHC class I pentamer staining and IFN-γ ELISPOT. These findings raise the possibility that autoantibody responses may collaborate with tumor-infiltrating T cells to influence clinical outcomes in EOC.

In addition to NY-ESO-1-specific autoantibodies, several factors expressed by ovarian tumors have shown a positive correlation with tumor-infiltrating T cells, including the chemokines CXCL9, CCL21, CCL22 [8], CCL2 and CCL5 [35]; p53 mutations [36]; and MHC class I [37]. Conversely, tumor-infiltrating T cells show a negative correlation with VEGF [8], B7-H1/PD-L1 [12], CD68+ macrophages [38] and the endothelin B receptor [39]. Thus, multiple factors influence the composition of tumor-infiltrating T cells in EOC.

While this study focused on a single, commonly recognized tumor antigen, NY-ESO-1, a large number of other autoantibody target antigens have been identified in EOC, including HOXA7, Ep-CAM, HSP-90, MUC-1 and p53 [23]–[25]. Indeed, tumor-specific autoantibody responses are common among EOC patients. For example, Stone et al. found that 44% of newly diagnosed EOC patients had an autoantibody response to at least 1 of a panel of 12 tumor antigens [24]. Future work will determine whether autoantibody responses to other tumor antigens also correlate with the presence of tumor-infiltrating T cells.

In contrast to serological responses, less is known about the target antigens of tumor-infiltrating T cells in EOC. T cell receptor (TCR) spectratyping studies have shown that tumor-infiltrating T cells in EOC represent oligoclonal populations, which is consistent with antigen-induced clonal expansion [40]–[44]. Many studies have shown recognition of autologous tumor cells by tumor-infiltrating T cells [45]–[50]. Moreover, previous work has identified T cells specific for HER2/*neu* [44], [51]–[53], p53 [54] and folate-binding protein [55], [56] among tumor-infiltrating or tumor-associated T cells. To our knowledge, the current study is the first to demonstrate recognition of NY-ESO-1 by tumor-infiltrating and tumor-associated (i.e., ascites derived) T cells, as evidenced by the results for patient IROC013. Intriguingly, T cell responses to NY-ESO-1 were undetectable in peripheral blood from this patient, indicating a strong enrichment of this T cell subpopulation in tumor and ascites. Indeed, the IFN-γ ELISPOT response to NY-ESO-1 was similar in magnitude to that seen with CEF control peptides. Nonetheless, the NY-ESO-1-specific subpopulation represented only 6.6% of all tumor-infiltrating CD8+ T cells in this subject, indicating there are likely many other antigens recognized by infiltrating T cells. Notably, over 80% of ovarian cancers exhibit loss of BRCA1 and/or BRCA2 function, leading to compromised DNA repair[57], [58]. One can speculate that the resulting genetic instability leads to the expression of abnormal proteins that may serve as neoantigens to the host immune system.

Although tumor infiltrating T cells are associated with prolonged progression-free and overall survival in EOC, it remains unclear whether this reflects an active or passive role of T cells. In other words, do T cells actively oppose tumor growth or are they simply markers of some other feature of the tumor that drives outcomes? There are several lines of evidence in support of the former possibility. First, not all T-cell subsets are associated with prognosis; rather, favorable outcomes are linked to the CD8+ subset and poor outcomes to the CD25+FoxP3+ subset [11], [12], [20], [21]. Second, several factors that are associated with active CD8+ cytolytic responses are also linked to favorable outcomes, including expression of IFN-γ [14], [15], the IFN-γ receptor [16] and IL-18 [17]. MHC class I has also been linked to increased survival in ovarian cancer [18], [19]. In the present study, which focused exclusively on the serous subtype, all evaluable tumors expressed MHC class I. Similar results have been reported by others for serous EOC [59], suggesting the vast majority of serous tumors are MHC class I positive and have the capacity to present antigen to tumor-infiltrating CD8+ T cells. Third, tumor-infiltrating T cells show significant cytotoxicity against ovarian tumors *in vitro* [43], [45], [47], [60], [61]. However, it is noteworthy that only a minority of tumors in this and another study [59] had significant Granzyme B-positive immune infiltrates, suggesting that cytotoxic function may be suppressed *in vivo*. And finally, as discussed above, tumor-infiltrating T cells show evidence of clonal expansion [40]–[44] and recognition of specific antigens [44], [51]–[56], as shown here for NY-ESO-1 (Figure 4). Thus, evidence is accumulating in favor of the concept that tumor-infiltrating T cells play an active role in promoting favorable clinical outcomes in EOC. Similarly, in colorectal cancer, functional markers associated with active Th1-like cytolytic T cell responses are linked to favorable clinical outcomes [62]. This suggests that immune modulatory strategies that enhance these naturally occurring T cell responses may improve clinical outcomes further.

In contrast to T cell infiltrates, the relationship between tumor-specific autoantibodies and clinical outcomes is less clear. This issue has been studied most extensively for the tumor antigen p53, which elicits autoantibody responses in 20–25% of EOC patients. Goodell et. al. reported a positive correlation between autoantibodies to p53 and increased overall survival in EOC [63]. However, other studies have found a negative correlation [64], [65] or no correlation [66], [67]. As for NY-ESO-1, we found a trend toward poor outcomes among patients with autoantibodies to NY-ESO-1 in the 35-case cohort studied here (data not shown), but this trend was not seen in an independent cohort of 35 patients from a prior study [24] (data not shown). As discussed above, the immune response to EOC involves multiple antigens, therefore we believe that the prognostic significance of autoantibodies is best addressed using an extended panel of tumor antigens and larger patient cohorts.

The autoantibody response to NY-ESO-1 correlated to T cell infiltration of tumor stroma as opposed to tumor epithelium. This may reflect a statistical issue, as the absolute number of T cells was higher in tumor stroma compared to epithelium, allowing the stromal values to achieve statistical significance. Indeed, Spearman rank correlation analysis showed a positive relationship between autoantibodies to NY-ESO-1 and infiltration of tumor epithelium by CD3+ and CD8+ cells, but this did not reach statistical significance (data not shown). Alternatively, there may be a biological explanation. In the Th1/Th2 paradigm, immune responses are thought to polarize toward humoral or cytolytic effector mechanisms [68]. Applied to our results, this would suggest that patients with strong autoantibody responses would have weak cytolytic responses, which may result in incomplete (i.e., stromal) infiltration of tumor tissue by T cells. A second possibility is suggested by murine models where autoantibody responses have been linked to weak CD8+ cytolytic T cell responses [69]. In this case, it was proposed that autoantibodies facilitate uptake and presentation of tumor antigens by B cells at the expense of dendritic cells. Since B cells are less potent antigen presenting cells than dendritic cells, the net result is an inferior T cell response against the tumor [69]. A final consideration is that, in the present study, autoantibody responses to NY-ESO-1 were correlated with infiltration of tumor by not only CD8+ T cells, but also by CD4+ and FoxP3+ T cells. The latter cells may represent regulatory T cells, which could inhibit cytolytic T cells responses and limit the extent of tumor infiltration [32]. We are currently collecting matched blood and tumor specimens from a larger, prospective cohort of EOC patients to better understand the relationship between tumor-specific autoantibodies, tumor-infiltrating T cells and clinical outcomes.

Although not the primary focus of this paper, we had the opportunity to assess the effect of neoadjuvant chemotherapy on tumor-infiltrating T cells in 15 patients. In general, treated tumors showed increased infiltration by all subsets of T cells, and this reached statistical significance for CD3+ and CD8+ cells. This is reminiscent of studies in breast cancer which showed increased T cell infiltration after chemotherapy with taxanes [70] and other agents [71] and an association with favorable clinical responses. Taxanes may also promote tumor immunity by enhancing T and NK cell activity [72]–[75] and antigen presentation [76]. Likewise, platinum agents can enhance cytokine synthesis by human T cells [77], abrogate suppressor T cell activity [78] and sensitize EOC cells to Fas-mediated apoptosis [79]. Thus, platinum/taxane-based chemotherapy may have favorable effects on host immunity to EOC, a hypothesis we are currently investigating in a prospective patient cohort.

Several groups have attempted adoptive immunotherapy of ovarian cancer using T cells expanded from tumor-infiltrating T cells [50], [80]–[89]. Although promising anecdotal responses have been reported, these efforts have generally met with limited success. It is noteworthy that solid tumor tissue from patient IROC013 was largely negative for expression of NY-ESO-1, despite having NY-ESO-1-specific T cells in tumor and ascites. If this scenario is representative of other EOC patients, it would suggest that many tumor-infiltrating T cells may recognize antigens that are poorly expressed by tumor tissue, possibly due to immune selection during tumor development. If we are to realize the promise of immunotherapy for EOC, there is a pressing need to identify target antigens that are essential to the growth and survival of recurrent, chemotherapy-resistant tumors. Our results suggest that autoantibody responses hold practical value for antigen identification and warrant further study with respect to their role in host tumor immunity and clinical outcomes.

## Materials and Methods

### Study subjects

All specimens and clinical data were obtained with informed consent under protocols approved by the Research Ethics Board of the BC Cancer Agency and the University of British Columbia. The retrospective case cohort consisted of 35 women with high-grade serous ovarian cancer from whom matched serum and tumor tissue was available (OvCaRe Ovarian Tumour Bank, Vancouver, BC, Canada). Tumor tissue was obtained at the time of primary surgery prior to any other treatment. Table 1 shows the general clinical characteristics of the 35-case cohort. The retrospective cohort also included tissue from an additional 15 women who received neoadjuvant chemotherapy prior to primary surgery; these cases are discussed separately in Results. Blood, ascites and tumor samples were also collected from a prospective cohort of 15 patients through the BC Cancer Agency's Tumour Tissue Repository. Control serum samples were obtained from 60 women with no known personal history of ovarian cancer or other cancers. All control subjects self reported receiving a negative mammographic result within the past year. The age distribution of the control cohort (mean 62.0 years, standard deviation 12.3 years, range 45.9 to 88.9 years) was similar to that of the case cohort.

### Tumor and serum specimens

#### Retrospective cohort

Tumor tissue was obtained during primary cytoreductive surgery. Tissue had an ischemia time of less than 30 minutes and spent less than 48 hours in formalin prior to being processed in paraffin. A tissue microarray (TMA) was constructed by taking duplicate 0.6 mm cores from tumor blocks after review of hematoxylin- and eosin-stained sections by a pathologist. Cores were selected from regions of tumor containing representative proportions of epithelium and stroma, while avoiding highly necrotic regions. TMAs were assembled using a Pathology Devices tissue arrayer (Westminster, MD). Serum samples from cases were collected prior to surgery or chemotherapy. Blood samples were processed by standard laboratory procedures.

#### Prospective cohort

Blood was collected prior to surgery in heparanized Vacutainer tubes, and peripheral blood mononuclear cells (PBMC) were isolated by Ficoll density centrifugation. HLA-A2 status was determined by flow cytometry on a FACSCalibur (BD Biosciences, San Jose, CA) after surface staining PBMC with anti-HLA-A2 antibody (clone BB7.2, BD Pharmingen, San Diego, CA). Ascites collected during surgery was centrifuged (1200 rpm for 10 min), and red blood cells (RBCs) were removed by treatment with ACK lysis buffer (Sigma, St. Louis, MO). Solid tumor removed during surgery was minced with scalpels to approximately 2 mm^2^ and was then digested overnight at 4°C in RPMI 1640 (Invitrogen, Carlsbad, CA) containing collagenase Type I and IV (each at 0.05 mg/ml), 0.025 mg/ml hyaluronidase and 0.01 mg/ml DNAse I (all from Sigma, St. Louis, MO). After digestion, material was passed through a 100 µm sterile cell strainer to remove clumps, and the resulting single-cell suspension was pelleted as described above.

### Immunohistochemistry and immunocytochemistry

TMAs were sectioned at 5 µm onto Superfrost plus slides (Fisher Scientific, Ottawa, ON) and incubated overnight at 37°C. Following deparaffinization, the slides were placed in a Ventana Discovery XT autostainer (Ventana, Tucson, AZ) for immunohistochemical staining. Ventana's standard CC1 protocol was used for antigen retrieval. Primary antibodies are listed in Table 2.

TMAs were incubated with primary antibodies for 60 minutes, and the appropriate cross-adsorbed, biotinylated secondary antibody (Jackson Immunoresearch, West Grove, PA) was applied for 32 minutes. Bound antibodies were detected using the DABMap kit (Ventana), counterstained with hematoxylin (Ventana), and coverslipped manually with Cytoseal-60 (Richard Allan, Kalamazoo, MI).

Ascites from serous ovarian cancer patients was collected and centrifuged using a Cytospin III cytocentrifuge (Thermo Shandon, Waltham, MA). The slides were then fixed in acetone and stored at −80°C until subjected to immunohistochemistry using standard protocols. The ascites sample from patient IROC013 was stained with antibodies to NY-ESO-1, CD3, CD8 and pan-cytokeratin (Table 2).

### Histopathological analysis

Immunostained TMAs were examined independently by two pathologists and a high degree of inter-observer concordance was achieved (r>0.7, p<0.0001) as well as intra-observer concordance on different sessions (r = 0.79, p<0.0001). Although the TMAs showed reasonable core retention, some cores were lost during the sectioning process and were not evaluable for one or more markers. Immunostaining was scored using three semi-quantitative IHC scoring systems. For NY-ESO-1 antigen, a modified H score approach was used [IHC score = (% positive neoplastic epithelial cells)×(staining intensity ranked from 0 to 3)] that ranged from 0–300. Positive was defined as an H score greater than 10; in practice, negative cases had H scores of 0–5, whereas positive cases had H scores ranging from 105–250. For MHC class I and II, a simplified four category scale was used (0 to 3+); a score of ≥2 was defined as positive. For immune cells, scoring was undertaken using a Chalkley 25 point array and methods similar to those used to assess vascular density. Briefly, each immunostained tumor was reviewed at low magnification and the core with the highest density of positive cells was selected. This core was then assessed at higher magnification (×20 objective) with a 25 cross hair grid overlaid on the image. Under a ×20 objective magnification, this grid defines an area of 0.56 mm^2^. The proportion of the core occupied by tumor epithelium was estimated, as was the total number of positive immune cells within the area of the grid. The number of grid points that coincided with positively staining immune cells within both epithelial and stromal areas was then determined. Positive immune cells that touched or overlapped with tumor epithelial compartments were counted as intraepithelial. All other positive cells were counted as intrastromal. For subsequent statistical analyses, an epithelial or stromal region was considered positive for a given immune cell population if there were more than five cells per unit area.

### ELISA to detect serum autoantibodies to NY-ESO-1

A cDNA encoding NY-ESO-1 was amplified by reverse-transcriptase PCR from the ovarian cancer cell line OVCAR-3. The C-terminal 25 amino acids were truncated to improve solubility of the protein. After sequencing, the cDNA was subcloned into the prokaryotic expression vector pDEST17, which adds a six-residue histidine tag at the N-terminus, and expressed in the *E. coli* strain BL21AI (Invitrogen, Carlsbad, CA). Urea-soluble recombinant NY-ESO-1 was purified on a HisTrap™ column (GE Healthcare, Fairfield Conn), eluted in urea buffer containing 500 mM imidazole, dialyzed in phosphate-buffered saline (PBS) and quantified by bicinchoninic acid (BCA) protein assay (Sigma, St. Louis, MO).

Maxisorp 96-well plates (Nunc, Roskilde, Denmark) were coated with 0.5 µg/well of purified NY-ESO-1 in 0.1 M carbonate buffer (33.5 mM Na_2_CO_3_, 0.1 M NaHCO_3_, pH 9.6) and incubated overnight at 4°C with gentle rocking. Plates were blocked with 3% bovine serum albumin (US Biological, Swampscott MA) in Tris buffered saline (TBS) containing 0.05% Tween-20 (3% BSA/TBST) for 2 hours at room temperature on a rapid shaker. All washes were performed with TBS/0.1% Tween-20 using a Skanwasher plate washer (Molecular Devices, Union City, CA). Plates were washed and incubated with patient and control serum diluted 1∶100 in 3% BSA/TBST for 1 hour at room temperature on a rapid shaker. All sera were assayed in triplicate. Plates were washed and incubated with goat anti-human IgG conjugated to horseradish peroxidase (Jackson, West Grove, PA) at 1∶10,000 in 3% BSA/TBST for 1 hour on a shaker at room temperature. Plates were developed with tetramethylbenzidine (TMB) (Neogen, Lansing, MI) for 3 minutes at room temperature and the reaction was stopped by addition of 1N HCl. The optical density of each well was analyzed at 450 nm on a Versamax plate reader (Molecular Devices, Union City, CA) and analyzed using Softmax Pro 4.8.

### IFN-γ ELISPOT analysis

ELISPOT plates (MSIP, Millipore, Billerica, MA) were pre-coated overnight with 10 µg/ml anti-IFN-γ capture antibody (1-D1K-Mabtech, Cincinnati, OH) and then blocked for 2 hours at 37°C with cRPMI (RPMI 1640, 10% FBS, 2 mM L-glutamine, 50 uM 2-mercaptoethanol, 10 mM HEPES, 10 mM MEM non-essential amino acids, 10 mM sodium pyruvate and 50 ug/ml gentamicin). Single cell suspensions of PBMC, ascites cells or solid tumor-derived cells were prepared in 10 ml cRPMI and plated in triplicate at 3×10^5^ cells per well. Cells were either left unstimulated (i.e., media only) or stimulated with HLA-A2-restricted tumor antigen peptides (NY-ESO-1_157–165_, p53_264–272_, WT-1_126–134_, HER-2/*neu*_654–662_; each at 10 µg/ml); a CEF (Cytomegalovirus, Epstein Barr, Flu) virus positive control peptide pool (10 µg/ml, Anaspec, San Jose, CA); or the T-cell mitogen phytohemagglutinin (PHA) (5 µg/ml). After overnight incubation at 37°C, ELISPOT plates were washed and incubated for 2 hours at 37°C with 1 µg/ml biotinylated anti-human IFN-γ (mAb 7-B6-1, Mabtech) followed by development with Vectastain ABC Elite kit and Vectastain AEC substrate reagent according to the manufacturer's instructions (Vector Labs, Burlingame, CA). Spots were quantified using a Zeiss automated ELISPOT reader and reported as the number of spot-forming cells (SFC) per 10^6^ PBMC.

### MHC class I pentamer analysis

Single-cell suspensions of ascites cells were depleted of red blood cells and stained with APC-conjugated HLA-A2-NY-ESO-1_157–165_ pentamer (Proimmune) according to the manufacturer's instructions. Pentamer staining was followed by surface staining with PerCP-conjugated anti-CD8 (53-6.7) (BD Pharmingen). Cells were analyzed on a BD FACSCalibur, and a minimum of 50,000 events were collected.

### Statistical analysis

Spearman correlation, Mann Whitney t-tests, and log rank tests were performed as appropriate to test statistical significance using Graphpad Prism v4.2 (Graphpad Software, San Diego, CA). Additional un-paired t-test analysis was performed using JMP statistical software (v7.0) (SAS Institute, Cary, NC).

## Acknowledgments

We thank the following for advice and technical assistance: Sindy Babinszky, Kristy Dillon, Xiaobo Duan, Sara Hahn, Jodi Leblanc, Heather Lockyer, Michele Martin, Erika Mehl, Jorg Michels, Melanie Olson, Lisa Szegedi, Dr. Nicholas van der Westhuizen, Erika Wall, Laura Walsh, Josh Wang, Nathan West, Taimei Yang and Siao Yong.

## Supporting Information

Table S1 (0.15 MB DOC)

Table S2 (0.13 MB DOC)
