# Supplementary material for: Tumor-Infiltrating T Cells Correlate with NY-ESO-1-Specific Autoantibodies in Ovarian Cancer
Source: PLoS One. 2008 Oct 15;3(10):e3409. doi: 10.1371/journal.pone.0003409 (PMC2561074; doi:10.1371/journal.pone.0003409)
Supplement: Table S1 — (0.15 MB DOC) [file pone.0003409.s001.doc]

Supplementary Table S1: Individual patient *scores for anti-NY-ESO-1 serum autoantibodies, NY-ESO-1 antigen, MHC Class I and II and lymphocyte markers.

| **Patient**  **ID #** | **αNY-ESO-1**  **Ab** | **NY-ESO-1**  **Ag** | **MHC**  **I** | **MHC**  **II** | ****%**  **Epithelium** | **CD3** | | | **CD8** | | | **CD4** | | | **CD20** | | |
| --- | --- | --- | --- | --- | --- | --- | --- | --- | --- | --- | --- | --- | --- | --- | --- | --- | --- |
|  |  |  |  |  |  | **Tot** | **ED** | **SD** | **Tot** | **ED** | **SD** | **Tot** | **ED** | **SD** | **Tot** | **ED** | **SD** |
| 427 | 9.81 | 25 | 3 | 0 | 0.90 | 20 | 2.2 | 19.6 | 30 | 1.1 | 39.2 | 14 | 1.1 | 19.6 | 7 | 0.0 | 19.6 |
| 421 | 9.14 | 100 | 3 | 2 | 0.94 | 40 | 10.6 | 49.0 | 60 | 11.7 | 16.3 | 17 | 4.3 | 16.3 | 0 | 0.0 | 0.0 |
| 479 | 9.0 | 0 | 3 | 1 | 0.88 | 50 | 4.6 | 89.8 | 60 | 11.4 | 40.8 | 35 | 3.4 | 40.8 | 5 | 1.1 | 8.2 |
| 369 | 8.01 | 40 | 2 | 0 | 0.82 | 50 | 6.1 | 27.2 | 35 | 1.2 | 21.8 | 20 | 0.0 | 21.8 | 1 | 0.0 | 5.4 |
| 297 | 7.73 | 0 |  |  | 0.24 | 15 | 16.8 | 0.0 | 35 | 4.2 | 6.6 | 1 | 0.0 | 1.3 | 0 | 0.0 | 0.0 |
| 337 | 7.72 | 20 | 3 | 3 | 0.92 | 70 | 15.2 | 134.8 | 60 | 2.2 | 24.5 | 20 | 1.1 | 36.8 | 10 | 1.1 | 12.3 |
| 382 | 7.42 | 60 | 3 | 3 | 0.94 | 90 | 15.0 | 62.7 | 100 | 17.1 | 31.3 | 30 | 5.3 | 47.0 | 2 | 1.1 | 0.0 |
| 488 | 4.80 | 0 | 3 | 2 | 0.79 | 60 | 2.5 | 57.0 | 70 | 1.3 | 23.8 | 75 | 0.0 | 42.8 | 40 | 0.0 | 33.3 |
| 388 | 2.61 | 0 |  |  | 0.33 | 20 | 6.0 | 4.5 | 10 | 3.0 | 1.5 |  | 0.0 | 0.0 | 0 | 3.0 | 0.0 |
| 419 | 1.11 | 0 | 3 | 3 | 0.96 | 10 | 1.0 | 23.5 | 3 | 1.0 | 0.0 | 0 | 0.0 | 0.0 | 0 | 0.0 | 0.0 |
| 515 | 0.81 | 0 | 3 | 0 | 0.92 | 10 | 0.0 | 12.3 | 3 | 1.1 | 0.0 | 4 | 1.1 | 0.0 | 0 | 0.0 | 0.0 |
| 307 | 0.69 | 0 |  |  | 0.78 | 150 | 3.9 | 138.1 | 65 | 5.2 | 8.9 | 20 | 2.6 | 4.5 | 100 | 1.3 | 106.9 |
| 374 | 0.62 | 5 | 3 |  | 0.86 | 3 | 0.0 | 7.0 | 0 | 0.0 | 0.0 | 0 | 0.0 | 0.0 | 0 | 0.0 | 0.0 |
| 296 | 0.53 | 0 |  |  | 0.47 | 70 | 6.4 | 18.8 | 35 | 4.3 | 5.6 | 6 | 4.3 | 1.9 | 2 | 0.0 | 1.9 |
| 394 | 0.39 | 0 | 3 | 1 | 0.71 | 25 | 4.2 | 6.9 | 10 | 2.8 | 3.4 | 4 | 2.8 | 0.0 | 3 | 1.4 | 0.0 |
| 209 | 0.18 | 0 | 3 | 1 | 0.21 | 80 | 4.7 | 22.9 | 60 | 0.0 | 15.2 | 15 | 0.0 | 3.8 | 18 | 0.0 | 7.6 |
| 496 | 0.05 | 0 | 2 | 2 | 0.77 | 10 | 0.0 | 12.8 | 5 | 1.3 | 4.3 | 40 | 0.0 | 21.4 | 0 | 0.0 | 0.0 |
| 432 | 0.01 | 0 | 3 |  | 0.63 | 40 | 3.2 | 10.7 | 30 | 8.0 | 2.7 | 35 | 12.8 | 2.7 | 6 | 0.0 | 18.7 |
| 390 | -0.09 | 0 |  | 3 | 0.11 | 35 | 9.4 | 3.4 | 10 | 9.4 | 2.2 | 1 | 0.0 | 1.1 | 4 | 0.0 | 1.1 |
| 385 | -0.01 | 0 | 3 | 2 | 0.69 | 70 | 2.9 | 42.5 | 50 | 1.4 | 19.6 | 25 | 0.0 | 16.3 | 4 | 0.0 | 6.5 |
| 384 | -0.12 | 0 |  |  | 0.80 | 70 | 12.6 | 29.4 | 60 | 6.3 | 24.5 | 50 | 5.0 | 19.6 | 8 | 2.5 | 0.0 |
| 321 | -0.24 | 0 | 3 | 0 | 0.80 | 60 | 10.1 | 39.2 | 60 | 1.3 | 34.3 | 9 | 1.3 | 0.0 | 0 | 0.0 | 0.0 |
| 540 | -0.27 | 0 | 3 | 2 | 0.69 | 60 | 10.2 | 12.7 | 50 | 11.7 | 3.2 | 20 | 1.5 | 6.4 | 5 | 1.5 | 0.0 |
| 229 | -0.33 | 0 |  |  | 0.70 | 90 | 5.7 | 33.6 | 60 | 1.4 | 20.1 | 40 | 5.7 | 26.9 | 9 | 1.4 | 0.0 |
| 331 | -0.37 | 0 | 3 | 3 | 0.94 | 20 | 2.1 | 16.3 | 5 | 1.1 | 16.3 | 2 | 0.0 | 16.3 | 0 | 0.0 | 0.0 |
| 376 | -0.59 | 0 | 3 | 3 | 0.84 | 25 | 1.2 | 6.1 | 5 | 1.2 | 6.1 | 11 | 2.4 | 0.0 | 2 | 1.2 | 0.0 |
| 378 | -0.61 | 0 | 3 | 3 | 0.10 | 5 | 9.8 | 0.0 | 0 | 0.0 | 0.0 | 0 | 0.0 | 0.0 | 0 | 0.0 | 0.0 |
| 332 | -0.66 | 0 | 3 |  | 1.00 | 10 | 1.0 |  | 2 | 1.0 |  | 4 | 1.0 |  | 1 | 1.0 |  |
| 300 | -0.71 | 0 | 3 | 2 | 0.63 | 15 | 1.6 | 5.3 | 3 | 0.0 | 2.7 | 4 | 1.6 | 0.0 | 0 | 0.0 | 0.0 |
| 342 | -0.74 | 0 | 3 | 3 | 0.96 | 50 | 7.3 | 24.5 | 20 | 2.1 | 49.0 | 60 | 3.1 | 122.5 | 4 | 0.0 | 24.5 |
| 327 | -0.90 | 0 | 3 | 3 | 0.91 | 50 | 9.8 | 11.8 | 40 | 4.4 | 0.0 | 12 | 1.1 | 23.5 | 2 | 1.1 | 0.0 |
| 325 | -0.99 | 0 | 3 | 3 | 0.94 | 30 | 7.5 | 65.3 | 35 | 4.3 | 16.3 | 5 | 0.0 | 32.7 | 3 | 1.1 | 16.3 |
| 319 | -1.04 | 0 |  |  | 0.89 | 20 | 3.4 | 9.4 | 5 | 0.0 | 9.4 | 0 | 0.0 | 0.0 | 2 | 0.0 | 9.4 |
| 217 | -1.16 | 0 | 3 | 3 | 0.92 | 10 | 2.2 | 0.0 | 8 | 2.2 | 0.0 | 0 | 0.0 | 0.0 | 0 | 0.0 | 0.0 |
| 443 | -1.20 | 0 | 3 | 2 | 0.75 | 10 | 2.7 | 4.0 | 5 | 1.3 | 4.0 | 1 | 0.0 | 4.0 | 0 | 0.0 | 0.0 |

*Autoantibody scores are reported as number of standard deviations from the mean of a cancer-free, age-matched control population; the thick line demarcates cases scored as positive (OD > 2) versus negative (OD <2). NY-ESO-1 antigen is reported as an H score; MHC Class I and II were scored on a scale of 0-3. For lymphocyte markers, “Tot” = Total number of cells per grid area (0.56μm^2^); “ED” = average number of cells per unit area of epithelium; “SD” = average number of cells per unit area of stroma.

**%Epithelium = percentage of tissue core consisting of epithelial tissue
