# Supplementary material for: Tumor-Infiltrating T Cells Correlate with NY-ESO-1-Specific Autoantibodies in Ovarian Cancer
Source: PLoS One. 2008 Oct 15;3(10):e3409. doi: 10.1371/journal.pone.0003409 (PMC2561074; doi:10.1371/journal.pone.0003409)
Supplement: Table S2 — (0.13 MB DOC) [file pone.0003409.s002.doc]

Supplementary Table S2: Individual patient *scores for anti-NY-ESO-1 serum autoantibodies and the functional markers FoxP3, CD25, TIA-1 and Granzyme B.

| **Patient**  **ID #** | **αNY-ESO-1**  **Ab** | **FoxP3** | | | **CD25** | | | **TIA-1** | | | **Granzyme B** | | |
| --- | --- | --- | --- | --- | --- | --- | --- | --- | --- | --- | --- | --- | --- |
|  |  | **Total** | **ED** | **SD** | **Total** | **ED** | **SD** | **Total** | **ED** | **SD** | **Total** | **ED** | **SD** |
| 427 | 9.81 | 8 | 0.0 | 19.6 | 1 | 0.0 | 9.8 | 8 | 2.2 | 9.8 | 3 | 1.1 | 0.0 |
| 421 | 9.14 | 35 | 1.1 | 65.3 | 6 | 1.1 | 0.0 | 30 | 3.2 | 16.3 | 25 | 1.1 | 32.7 |
| 479 | 9.00 | 60 | 1.1 | 73.5 | 10 | 2.3 | 16.3 | 55 | 8.0 | 24.5 | 20 | 0.0 | 32.7 |
| 369 | 8.01 | 15 | 0.0 | 10.9 | 3 | 0.0 | 5.4 | 15 | 1.2 | 10.9 | 0 | 0.0 | 0.0 |
| 297 | 7.73 | 15 | 0.0 | 2.6 | 7 | 25.2 | 3.9 | 5 | 4.2 | 1.3 | 0 | 0.0 | 0.0 |
| 337 | 7.72 | 40 | 1.1 | 24.5 | 18 | 3.3 | 0.0 | 40 | 3.3 | 12.3 | 0 | 0.0 | 0.0 |
| 382 | 7.42 | 15 | 3.2 | 15.7 | 4 | 1.1 | 0.0 | 60 | 12.8 | 0.0 | 0 | 0.0 | 0.0 |
| 488 | 4.80 | 60 | 0.0 | 33.3 | 30 | 0.0 | 28.5 | 45 | 0.0 | 19.0 | 20 | 6.3 | 4.8 |
| 388 | 2.61 | 5 | 0.0 | 1.5 |  | 0.0 | 0.0 |  | 0.0 | 0.0 | 0 | 0.0 | 0.0 |
| 419 | 1.11 | 2 | 0.0 | 23.5 | 3 | 0.0 | 23.5 | 7 | 1.0 | 0.0 | 8 | 1.0 | 47.0 |
| 515 | 0.81 | 2 | 1.1 | 0.0 | 2 | 1.1 | 0.0 | 3 | 0.0 | 12.3 | 1 | 0.0 | 12.3 |
| 307 | 0.69 | 50 | 3.9 | 17.8 | 5 | 1.3 | 0.0 | 15 | 1.3 | 4.5 | 1 | 1.3 | 0.0 |
| 374 | 0.62 | 1 | 0.0 | 7.0 | 0 | 0.0 | 0.0 | 2 | 1.2 | 7.0 | 2 | 1.2 | 0.0 |
| 296 | 0.53 | 10 | 4.3 | 0.0 | 6 | 4.3 | 0.0 | 23 | 8.6 | 0.0 | 1 | 0.0 | 1.9 |
| 394 | 0.39 | 8 | 1.4 | 3.4 | 3 | 1.4 | 0.0 | 3 | 1.4 | 0.0 | 1 | 1.4 | 0.0 |
| 209 | 0.18 | 15 | 0.0 | 5.1 | 6 | 0.0 | 1.3 | 20 | 4.7 | 2.5 | 2 | 0.0 | 1.3 |
| 496 | 0.05 | 15 | 0.0 | 17.1 | 10 | 0.0 | 8.6 | 3 | 0.0 | 4.3 | 0 | 0.0 | 0.0 |
| 432 | 0.01 | 20 | 3.2 | 5.3 | 13 | 1.6 | 2.7 | 30 | 3.2 | 5.3 | 4 | 0.0 | 2.7 |
| 390 | -0.09 | 5 | 9.4 | 0.0 | 1 | 0.0 | 1.1 | 10 | 9.4 | 0.0 | 5 | 0.0 | 1.1 |
| 385 | -0.10 | 20 | 0.0 | 9.8 | 8 | 2.9 | 3.3 | 30 | 1.4 | 3.3 | 20 | 0.0 | 6.5 |
| 384 | -0.12 | 40 | 6.3 | 4.9 | 21 | 3.8 | 4.9 | 25 | 2.5 | 4.9 | 3 | 0.0 | 4.9 |
| 321 | -0.24 | 5 | 0.0 | 9.8 | 2 | 1.3 | 0.0 | 50 | 3.8 | 29.4 | 15 | 1.3 | 4.9 |
| 540 | -0.27 | 15 | 4.4 | 0.0 | 13 | 1.5 | 3.2 | 50 | 5.8 | 15.9 | 7 | 1.5 | 0.0 |
| 229 | -0.33 | 60 | 2.9 | 6.7 | 20 | 8.6 | 10.1 | 45 | 0.0 | 10.1 | 2 | 0.0 | 3.4 |
| 331 | -0.37 | 2 | 0.0 | 16.3 | 1 | 1.1 | 0.0 | 3 | 1.1 | 16.3 | 0 | 0.0 | 0.0 |
| 376 | -0.59 | 15 | 2.4 | 18.4 | 9 | 2.4 | 0.0 | 12 | 1.2 | 6.1 | 0 | 0.0 | 0.0 |
| 378 | -0.61 | 3 | 0.0 | 1.1 | 1 | 0.0 | 1.1 | 2 | 9.8 | 0.0 | 0 | 0.0 | 0.0 |
| 332 | -0.66 | 2 | 1.0 |  | 5 | 1.0 |  | 8 | 1.0 |  | 3 | 1.0 |  |
| 300 | -0.71 | 15 | 0.0 | 5.3 | 12 | 1.6 | 2.7 | 7 | 1.6 | 2.7 | 0 | 0.0 | 0.0 |
| 342 | -0.74 | 5 | 1.0 | 0.0 | 5 | 1.0 | 0.0 | 12 | 3.1 | 0.0 | 0 | 0.0 | 0.0 |
| 327 | -0.90 | 20 | 2.2 | 0.0 | 2 | 1.1 | 0.0 | 22 | 4.4 | 23.5 | 0 | 0.0 | 0.0 |
| 325 | -0.99 | 20 | 1.1 | 16.3 | 13 | 2.1 | 16.3 | 32 | 1.1 | 32.7 | 0 | 0.0 | 0.0 |
| 319 | -1.04 | 10 | 0.0 | 18.8 | 20 | 2.2 | 9.4 | 13 | 1.1 | 0.0 | 0 | 0.0 | 0.0 |
| 217 | -1.16 | 3 | 0.0 | 12.3 | 1 | 1.1 | 0.0 | 5 | 1.1 | 0.0 | 0 | 0.0 | 0.0 |
| 443 | -1.20 | 0 | 0.0 | 0.0 | 0 | 0.0 | 0.0 | 5 | 1.3 | 0.0 | 4 | 1.3 | 0.0 |

* Autoantibody scores are reported as number of standard deviations from the mean of a cancer-free, age-matched control population; the thick line demarcates cases scored as positive (OD > 2) versus negative (OD <2). For lymphocyte markers, “Total” = Total number of cells per grid area (0.56μm^2^); “ED” = average number of cells per unit area of epithelium; “SD” = average number of cells per unit area of stroma.
